# Promising Outcomes in Acromegaly Patients Receiving CyberKnife Stereotactic Hypofractionated Radiotherapy

**DOI:** 10.7759/cureus.47936

**Published:** 2023-10-29

**Authors:** Rasim Meral, Ozlem S Selcukbiricik, Ayse K Uzum, Serdar Sahin, Murat Okutan, Mehmet Barburoglu, Ilyas Dolas, Musa Altun, Sema Yarman, Pinar Kadıoglu

**Affiliations:** 1 Department of Clinical Oncology, Istanbul University Institute of Oncology, Istanbul, TUR; 2 Department of Radiation Oncology, Istanbul University School of Medicine, Istanbul, TUR; 3 Department of Endocrinology, Istanbul University School of Medicine, Istanbul, TUR; 4 Department of Endocrinology, Istanbul University - Cerrahpasa School of Medicine, Istanbul, TUR; 5 Department of Medical Physics, Istanbul University Institute of Oncology, Istanbul, TUR; 6 Department of Radiology, Istanbul University School of Medicine, Istanbul, TUR; 7 Department of Neurological Surgery, Istanbul University School of Medicine, Istanbul, TUR

**Keywords:** radiation induced optic neuropathy, pituitary insufficiency, pituitary adenoma, endocrine remission, cyberknife stereotactic hypofractionated radiotherapy, acromegaly

## Abstract

Background: The primary treatment for patients with acromegaly has traditionally been transsphenoidal surgery, with decreasing reliance on radiotherapy (RT) due to advancements in pharmacotherapy (PT). Despite these advancements, a substantial portion of patients still face persistent acromegaly, necessitating novel treatment approaches. This study investigates the role of CyberKnife Stereotactic Hypofractionated Radiotherapy (CK-HFRT) in persistent acromegaly.

Objective: The primary objective was to assess the impact of CK-HFRT on endocrine remission (ER) rates while maintaining acceptable toxicity levels.

Methods: The study retrospectively analyzed 31 consecutive patients with acromegaly who received CK-HFRT following multiple unsuccessful surgeries and prolonged PT without ER. Various CK-HFRT dose fractionation regimes were administered, and dose volume histograms were evaluated. Tumor control, cured disease (CD), endocrine remission (ER) rates, and overall survival were estimated at a median follow-up of 62 months. Acute and late toxicity, including pituitary insufficiency and radiation-induced optic neuropathy (RION), were also assessed.

Results: At 62 months of follow-up, the study group demonstrated excellent tumor control with 100% nonprogressive adenomas. Endocrine remission was achieved in 86.7% of patients, with a 22.4% CD rate at five years. Pituitary insufficiency occurred in 32.3% of patients, and no cases of RION were reported. The study observed three deaths related to cardiovascular diseases, all in patients receiving PT. Overall survival at five years was 79.2%.

Conclusion: CyberKnife stereotactic hypofractionated radiotherapy, as an adjunct to PT, provides a viable treatment option for patients with persistent acromegaly following unsuccessful surgeries. The therapy results in substantial ER rates and tumor control while minimizing the risk of permanent radiation-induced optic neuropathy. However, the decision to administer CK-HFRT should be individualized, considering the patient's overall condition and treatment history.

## Introduction

The first-line treatment of acromegaly is transsphenoidal surgery (89%), followed by pharmacotherapy (PT) and radiotherapy (RT). From the 20th to 21st century, the use of RT for patients with acromegaly decreased from 45% to 15%. However, in the same period, disease control has also decreased from 72% to 68% [1]. Another interesting trend observed in parallel with the decrease in RT use in patients with acromegaly has been the increasing percentage of patients in surveillance without any treatment [2]. These trends are compelling to reevaluate the role of RT in the modern treatment of patients with acromegaly.

Cancer registries indicate that around 55% of patients with acromegaly achieve a cure with the current treatment approach. However, approximately 45% of patients still have persistent acromegaly, where the pre-treatment growth hormone (GH) level serves as the major determinant of prognosis [3]. Therefore, there is an inevitable need for novel treatment strategies to improve the outcomes of patients with persistent acromegaly. These strategies may include debulking surgery or maximal safe resection (MSR), preoperative PT, and early RT during the disease.

Patients with acromegaly who have residual mass after initial MSR may benefit from PT and/or RT [4]. Preliminary results have shown that endocrine remission (ER) rates doubled with MSR. Even though total resection could not be achieved, extensive subtotal resection in patients with acromegaly would increase ER probability with PT (67.1% vs. 89.45%; p < 0.05), and 20.8% (25/120) of patients were also treated with additional stereotactic radiosurgery (SRS) who have been accepted to have resistant acromegaly if ER could not be achieved with postsurgical PT for one year [5]. Preoperative PT would allow a pituitary surgeon to perform a more extensive resection of the tumor. Pharmacotherapy can also be instrumental in the postoperative period. Nevertheless, the use of RT is limited due to its associated side effects, making a treatment option of last resort to achieve a CD. Therefore, a meticulous evaluation is needed to determine whether the benefits of RT outweigh the disadvantages [6]. In this study, we report the contribution of high-precision RT as CyberKnife stereotactic hypofractionated radiotherapy (CK-HFRT) to outcomes of patients with persistent acromegaly within a multidisciplinary approach.

Early outcomes of this study have been previously presented partly as a meeting abstract on September 25-28, 2016, at the American Society for Radiation Oncology Annual Meeting.

## Materials and methods

We retrospectively reviewed the medical charts of 31 consecutive patients with growth hormone-secreting pituitary adenomas who were treated with CyberKnife stereotactic hypofractionated radiotherapy (CK-HFRT) in addition to multiple surgeries and prolonged PT between January 2011 and December 2015. All the patients were diagnosed as acromegaly except one with gigantism. They gave informed written consent for treatment, analysis, and publication of treatment data. The patients were also asked to self-assess their response to CK-HFRT. The patients or close relatives of three patients who died also gave verbal consent for the publication of their treatment outcomes. The study was approved by Istanbul University, Institute of Oncology Academic Coordination Board (2023/1016). 

Management of patients with acromegaly

We diagnosed acromegaly biochemically with elevated age and sex-adjusted serum insulin-like growth factor-I (IGF-I) levels and/or growth hormone (GH) levels that are not suppressed in the oral glucose tolerance test. A pituitary MRI was performed to determine adenoma size and location. Primary therapy was maximal safe resection generally with a transsphenoidal approach as recommended by Endocrine Society. Patients with persistent (incomplete resection) acromegaly have been treated usually with somatostatin receptor ligands (SRL), dopamine agonists (DA) for mild disease, and with pegvisomant when the disease did not respond to SRL. In case of partial response to maximal doses of these drugs combination therapy can be considered. If PT had been ineffective or intolerable, SRT was considered [7].

CyberKnife stereotactic hypofractionated radiotherapy

**Figure 1:**
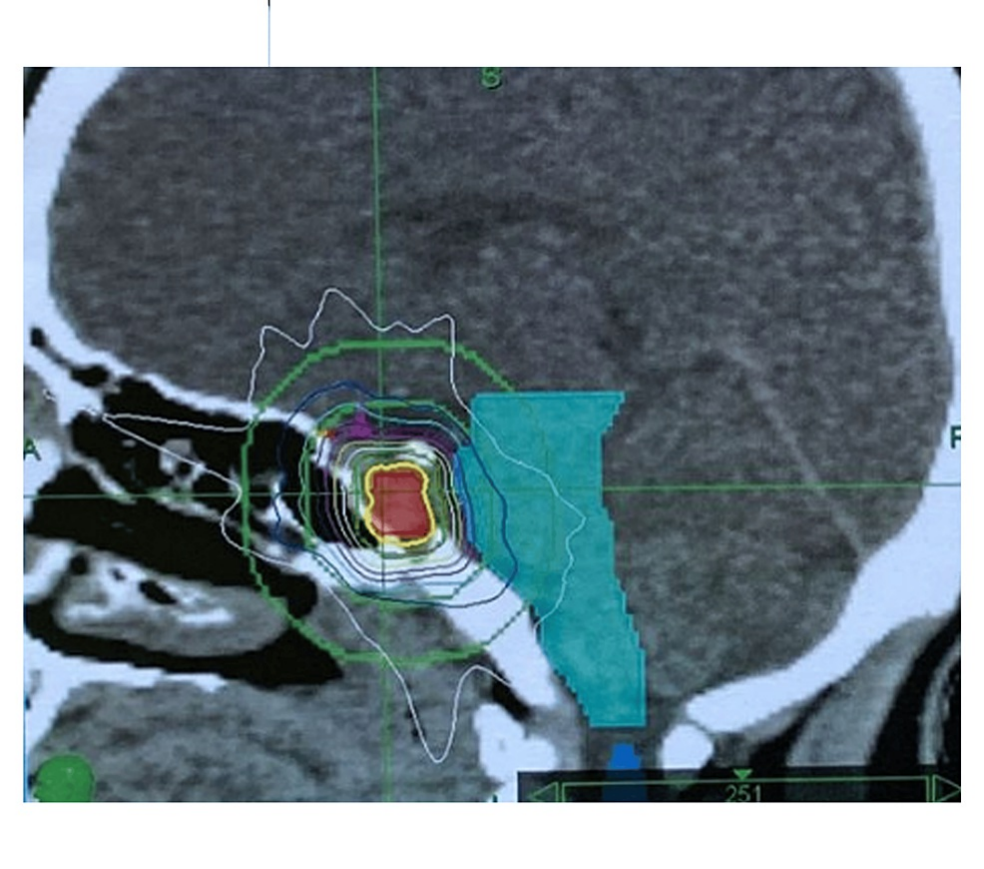
Dose distribution map of CyberKnife (sagittal plane) In this patient with persistent acromegaly, the 21 Gy total dose in 3 daily 7 Gy fractions to the planning target volume was prescribed to 94% isodose line with 96.55% coverage, while optic chiasm could be protected below 13.65 Gy maximum total dose. The sagittal view shows the optic pathway out of the high-dose region.

**Figure 2:**
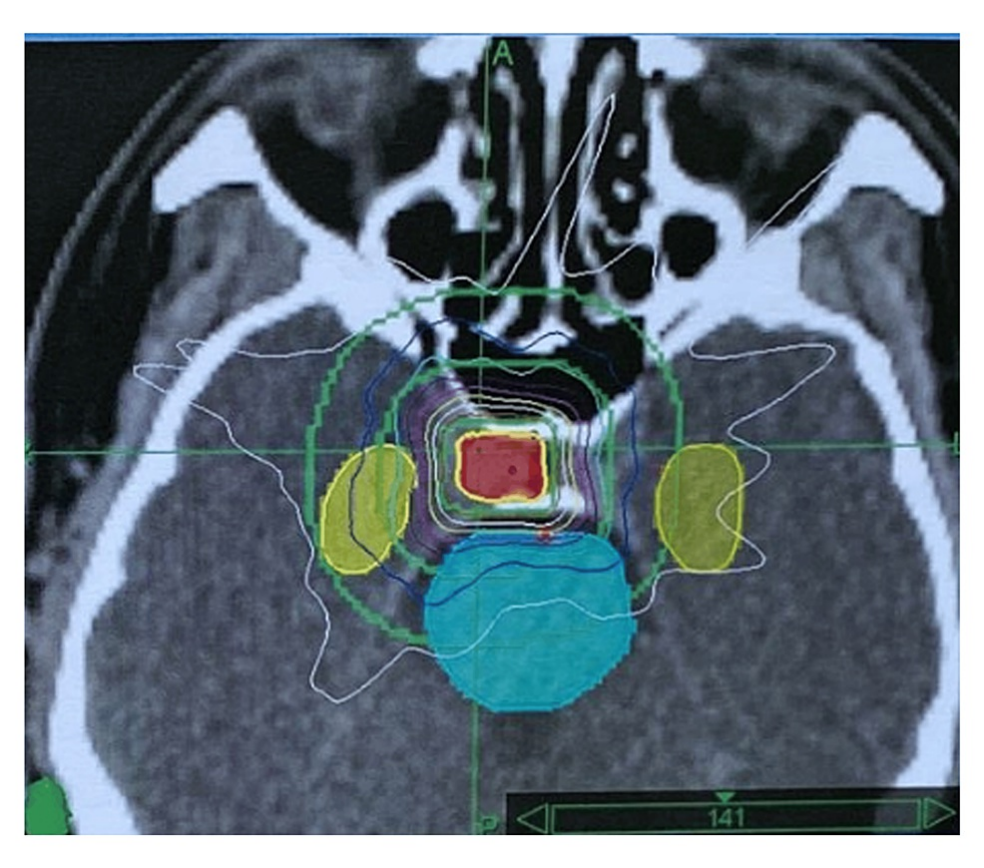
Dose distribution map of CyberKnife (axial plane) In this patient with persistent acromegaly, the 21 Gy total dose in 3 daily 7 Gy fractions to the planning target volume was prescribed to 94% isodose line with 96.55% coverage. The axial view shows the hippocampuses and the brain stem out of the high-dose region.

**Figure 3:**
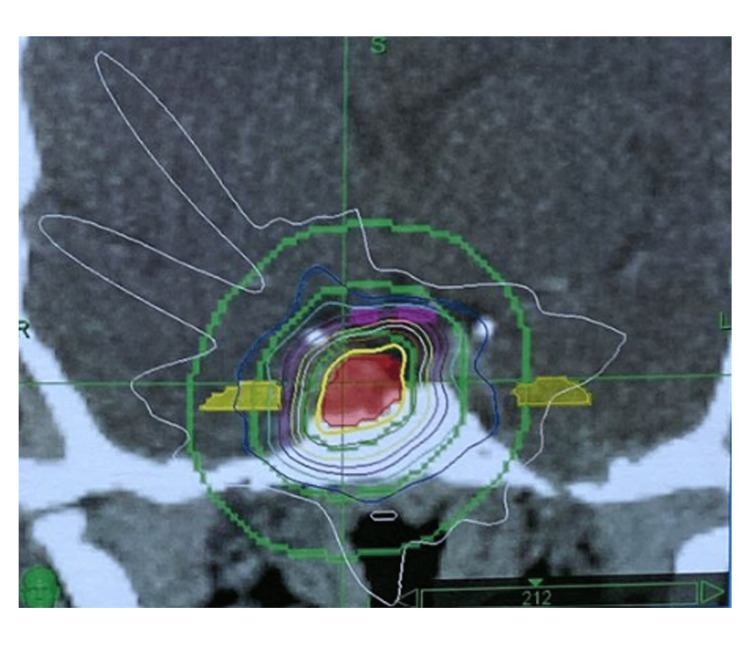
Dose distribution map of CyberKnife (coronal plane) In this patient with persistent acromegaly, the 21 Gy total dose in 3 daily 7 Gy fractions to the planning target volume was prescribed to 94% isodose line with 96.55% coverage. The coronal view shows the hippocampuses and the optic chiasm out of the high-dose region.

Fifteen (48.4%) patients received a total dose of 21 Gy in three daily fractions and seven (22.6%) 25 Gy in five daily fractions when the residual pituitary adenoma was > 2 mm away from the optic pathway. Four (12.9%) patients were irradiated with 30 Gy in 10 daily fractions with residual adenomas invading both cavernous sinuses and/or ≤ 2 mm close to the optic pathway. Five (16.1%) patients who have been treated with RT or SRS before received a total dose of 20 Gy in five daily fractions (Table 1). The dose was prescribed to a median 88% (75% - 94%) isodose line (Table 2 and Figures 1-3). 

**Table 1:** CyberKnife stereotactic hypofractionated radiotherapy dose/fractionation schemes of the patients with acromegaly *Patients treated with radiotherapy or radiosurgery before; BED: Biologically effective dose; α/β: Alpha/Beta; EQD2: 2 Gy equivalent dose; Gy: Gray; fx: Fraction.

	BED (α/β=4)	EQD_2_(α/β=4)	N	%
21 Gy / 3 fx	57.75 Gy	38.50 Gy	15	48.4
25 Gy / 5 fx	56.25 Gy	37.50 Gy	7	22.6
30 Gy /10 fx	52.50 Gy	35.00 Gy	4	12.9
20 Gy / 5 fx*	40.00 Gy	26.67 Gy	5	16.1

**Table 2:** Indexes and coverage in the CyberKnife stereotactic hypofractionated radiotherapy plans of patients with acromegaly &: Interquartile Range; *: The dose was prescribed to median 88% (75% to 94%) isodose line; CI: Conformity index; nCI: New conformity index; HI: Homogeneity index.

Index	Median	IQR^&^
CI	1.42	0.26
nCI	1.44	0.25
HI	1.14	0.09
Coverage (%) *	97.06	2.12

We calculated Biologically Effective Dose (BED) and Equivalent Dose 2 Gy (EQD2) with an α/β ratio of 4 as adenomas responded late to ionizing radiation as follows: BED = nd (1 + d/(α/β)) and EQD2 = BED/(1 + 2/(α/β)) where ‘n’ is the number of irradiation fractions and ‘d’ is the dose per irradiation fraction [8]. Planning target volume (PTV) was defined as the adenoma gross tumor volume (GTV) with a 0-1 mm margin. If the adenoma could not be localized, PTV was defined as the pituitary gland and the cavernous sinuses. Median PTV volume was calculated at 3486 mm^3^ (1311.5 mm^3^ - 6307.0 mm^3^). Dose-volume histograms of the PTV and optic pathway were evaluated. All the patients were given CK-HFRT median 51 (24- 150) months after several surgeries and MT without achieving ER.

Statistical analyses

Tumor control was defined as nonprogressive adenoma or adenoma with objective response (complete or partial), CD as normal IGF-I level without PT, ER as normal IGF-I level with PT, and uncontrolled disease as high IGF-I level with PT. After a median of 62 (IQR: 38 - 96) months of follow-up, the addition of CK-HFRT to PT, CD, and ER one minus survival and overall survival (OS) rates were calculated with the Kaplan-Meier method. Endocrine remission rates with different BED levels were compared with crosstabs Pearson Chi-Square Test. Endocrine insufficiency and RION related to CK-HFRT have been evaluated.

## Results

The median age of the study group was 47 (37 - 58) years and female to male ratio was 1.38. Optic neuropathy has been found in 12 (38.7%) of 31 patients before CK-HFRT. Surgery was performed with a trans-sphenoidal (TS) approach in 29 (93.5%) patients, craniotomy in one (3.2%) patient and one (3.2%) patient refused surgery. A second surgery was performed in nine (29%) patients. Pharmacotherapy was given following initial surgery or diagnosis in one patient without surgery for a median of 51 (IQR: 24 - 150) months without achieving ER in all 31 patients with acromegaly until the addition of CK-HFRT to PT. The indication for CK-HFRT was prolonged PT following surgery without ER in 24 (77.4%), high risk of surgery in three (9.7%), and progression of the adenoma, refusal of surgery, fibrosis preventing further surgery and unresponsiveness to PT each in one (3.2%) patient (Table 3).

**Table 3:** Patient characteristics (N = 31). CK-HFRT: CyberKnife stereotactic hypofractionated radiotherapy; IQR: Interquartile range.

Characteristic	
Age (years) median (IQR)	47 (37-58)
Gender (female/male)	1.38
Diagnosis	
Acromegaly	30 (96.8%)
Gigantizm	1 (3.2%)
Type of surgery	
Trans-sphenoidal	29 (93.5%)
Craniotomy	1 (3.2%)
None	1 (3.2%)
Second surgery	
Trans-sphenoidal	9 (29%)
Time (months) from Initial surgery to CK-HFRT median (IQR)	51 (24-150)
CK-HFRT indications	
Prolonged pharmacotherapy	24 (77.4%)
High risk of surgery	3 (9.7%)
Refusal of surgery	1 (3.2%)
Progression under pharmacotherapy	1 (3.2%)
Intolerance to pharmacotherapy	1 (3.2%)
Second surgery cannot be applied due to fibrosis	1 (3.2%)
Optic Neuropathy Before CK-HFRT	12/31 (38.7%)
Diameter (mm) of target volume median (IQR)	15 (10-22)
Planning target volume (mm^3^) median (IQR)	3486 (1311.5 – 6307.0)

**Figure 4:**
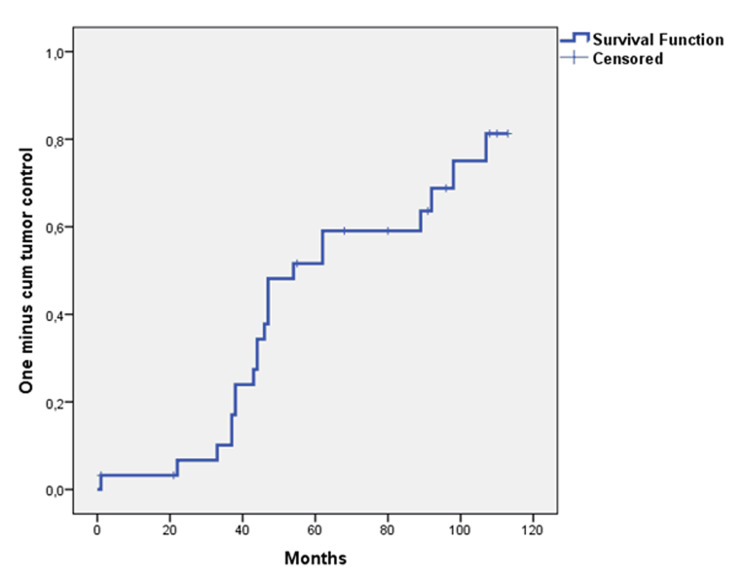
Probability of tumor control Probability of objective (partial or complete) tumor control following CyberKnife stereotactic hypofractionated radiotherapy with or without pharmacotherapy in 31 patients with acromegaly with Kaplan-Meier method 1 minus cumulative survival estimate: 51.8% at 5-years (median follow-up: 62 months (38 -96 months)).

**Figure 5:**
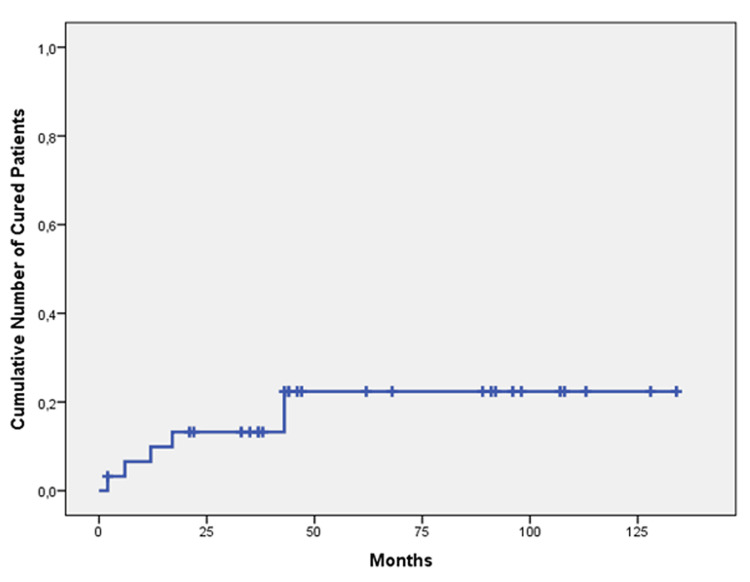
Probability of patients with cured disease Probability of patients with cured disease following CyberKnife stereotactic hypofractionated radiotherapy in 31 patients with acromegaly with Kaplan-Meier methods 1 minus cumulative survival estimate: 22.4% at five years (median follow-up: 62 months (38 – 96 months)).

**Figure 6:**
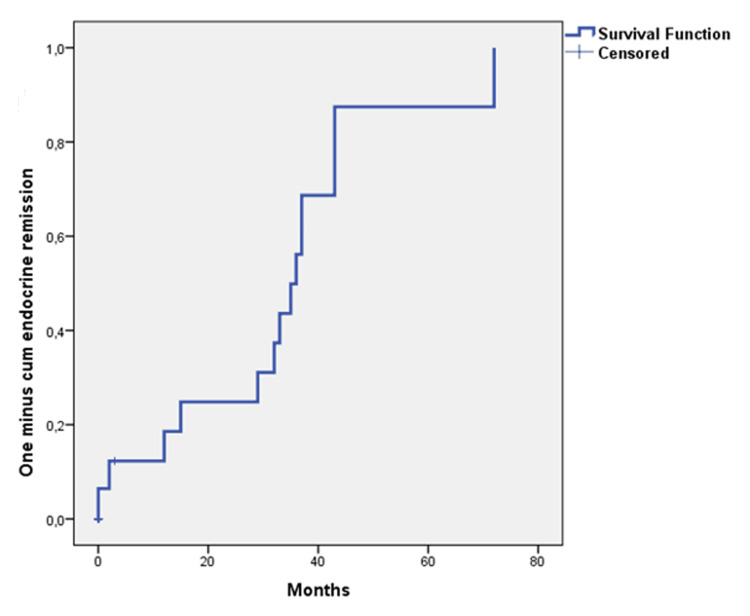
Probability of patients with endocrine remission Probability of patients with endocrine remission with pharmacotherapy following CyberKnife stereotactic hypofractionated radiotherapy in 31 patients with acromegaly with Kaplan-Meier methods 1 minus cumulative survival estimate: 86.7% in 5 years (median follow-up: 62 months (IQR: 38 - 96 months)).

At 62 months of median follow-up crude tumor control (patients without tumor progression) has been calculated 100% with four (12.9%) complete responses, 17 (54.8%) partial responses, two (6.5%) minimal regression, and eight (25.8%) stable disease. Six out of 31 (19.4%) patients had CD and 20 out of 31 (64.5%) patients had ER. Endocrine remission was achieved in 26 of 31 (83.9%) patients. Five out of 31 (16.1%) patients had uncontrolled disease when the analysis was done. In 31 patients with acromegaly at two and five years following the addition of CK-HFRT to PT Kaplan-Meier estimated objective TC rates were 6.7% and 51.8%, CD rates were 13.2% and 22.4%, ER rates were 46.9% and 86.7%, respectively (Table 4 and Figures 4-6). Endocrine remission and CD rates with BED levels for α/β = 4 Gy were not significantly different compared with crosstabs Pearson Chi-Square Test (2-sided p = .945).

**Table 4:** Results of CyberKnife stereotactic hypofractionated radiotherapy (CK-HFRT) for patients with acromegaly PR: Partial response; CR: Complete response (tumor control at 47 months median follow-up was 100%).

	Two years (%)	Five years (%)
Tumor control (PR and CR)	6.7	51.8
Cured disease following CK-HFRT	13.2	22.4
Endocrine remission with continuing pharmacotherapy following CK-HFRT	46.9	86.7
Overall survival	-	79.2

Acute side effects were limited to temporary syncope, imbalance, and local hair loss each in one patient. Ten (32.3%) patients developed pituitary insufficiency due to CK-HFRT. Optic neuropathy was detected in nine (29%) patients before CK-HFRT of which three (9.7%) improved following irradiation. None of the patients developed RION (Table 5). 

**Table 5:** Side effects of CyberKnife stereotactic hypofractionated radiotherapy.

Time	Type of side effect	N (%)
Early (temporary)		
	Syncope	1 (3.2%)
	Imbalance	1 (3.2%)
	Local hair loss	1 (3.2%)
Late (permanent)		
	Pituitary insufficiency	10 (%32.3)
	Radiation induced optic neuropathy	0 (%0.0)

Twenty-five (80.6%) patients thought their health had improved following CK-HFRT, while three (9.7%) did not observe any difference in their health status with CK-HFRT, and three (9.7%) patients died (Table 6).

**Table 6:** Self-assessment of patients with acromegaly of their response to CyberKnife stereotactic hypofractionated radiotherapy at median 62 months (IQR: 38 - 96) follow-up.

Self-assessment of the patient	N	%
I feel better following radiotherapy.	25	80.6
I don’t feel any difference in my health following radiotherapy.	3	9.7
My health is worse following radiotherapy.	0	0.0
Death	3	9.7

**Figure 7:**
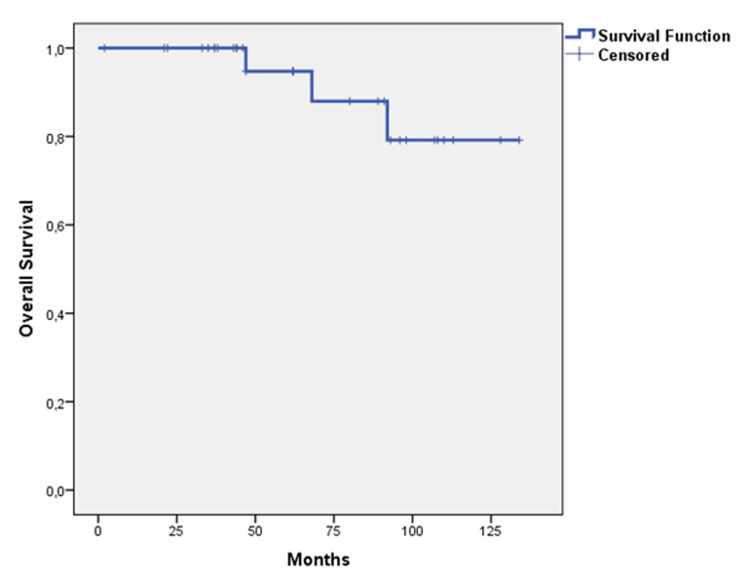
Probability of overall survival The probability of overall survival following CyberKnife stereotactic hypofractionated radiotherapy in 31 patients with acromegaly with Kaplan-Meier method survival was estimated as 79.2% at five years. Median time was not reached at the time of the analysis (mean 131.260 months; 95% CI: 108.163 – 134.357).

All three patients died because of cardiovascular disease long after CK-HFRT at 47, 68, and 92 months of their follow-up. These three patients who died because of cardiac disease were all on medical treatment, of whom one had uncontrolled disease. The probability of OS following CK-HFRT in 31 patients with acromegaly with Kaplan-Meier method survival estimate at five years was 79.2% (mean 131.260 months; 95% CI: 108.163 - 134.357) (Figure 7).

## Discussion

In our patients with persistent acromegaly who had been treated with CyberKnife stereotactic hypofractionated radiotherapy high tumor control and endocrine remission rates have been achieved. However, the cured disease rate remained relatively low. The high endocrine remission rate of 86.7% in patients with persistent acromegaly reflects the success of a multidisciplinary approach with improved surgical techniques, precise radiotherapy, and most of all powerful suppressive drug treatments applied by endocrinology discipline and considerably longer follow-up of a median of 62 months. In addition, we applied CyberKnife stereotactic hypofractionated radiotherapy relatively early with medical treatment and not as a last choice in the treatment of patients with persistent acromegaly.

There were several limitations encountered that warrant consideration in the interpretation of our retrospective study. First of all, a relatively small sample size (31 patients) might limit the generalizability of the findings and lower the statistical power of the study. Patients in the study had undergone various surgeries and PT before receiving CK-HFRT. It was challenging to decide the causality between different treatments and study outcomes. Additionally, a range of treatment dosages (21 Gy/3 fx, 25 Gy/5 fx, 30 Gy/10 fx, and 20 Gy/5 fx) with varying fractionation schedules were used in CK-HFRT. This variability could influence time to ER following CK-HFRT and makes it challenging for direct comparisons with other studies reporting on RT dose-response relations. Furthermore, selection bias was also a concern, as the patients with persistent acromegaly treated with CK-HFRT had the most unfavorable prognosis. Despite these limitations, our study provides valuable insights into the RT of patients with persistent acromegaly.

The remission rates of patients with acromegaly have increased with the advent of novel drugs. However, cure rates remained low. Minniti et al. have reported CD rates of 9% at two years and 29% at five years, respectively [9]. In general, studies have shown that following either conventional or conformal RT in patients with acromegaly CD rates would increase in time approximately from 10% to 25% and ER rates from 38% to 75% at two and five years, respectively [10]. Nevertheless, it should be noted that conventional RT cohorts have considerably longer follow-up periods, and the CD rates following RT can be as high as 74% after 15 years in patients with acromegaly. At this point, it is unknown whether the same CD rates can be achieved with CK-HFRT. In our cohort, the CD rate was 22.4% at five years and median follow-up was relatively short (62 months) compared to conventional RT cohorts.

A systematic review and meta-analysis of pituitary adenomas treated with Gamma Knife stereotatic radiosurgery (GK-SRS) of 20 to 28 Gy to the 50% isodose line in 13 studies were on patients with acromegaly. Five-year estimates of ER and Progression Free Survival (PFS) were calculated as 46% (range 50%-65%) and 52% (range 20%-73%), respectively [11]. Pre-irradiation hormone levels and the use of SRL and SRS doses have been the main factors predicting treatment outcomes in patients with acromegaly. Studies have suggested that the marginal dose of SRS in patients with acromegaly should be around 20 Gy to 25 Gy. Of note, various studies have reported that single fraction marginal doses ranging from 18 Gy to 32 Gy did not show significant differences in ER rates or time to ER. Pollock et al. reported a 50% CD rate at a median 36-month follow-up, 11% at two years, and 60% at five years actuarial rates of CD, respectively. Endocrine remission rate was found to be significantly higher in patients with IGF-I <2.25 nl/mL compared with patients with IGF-I >2.25 nl/mL and in patients with cessation of octreotide (SRL) before and during RT compared with patients irradiated without cessation of octreotide. They suggest discontinuation of SRL starting one month before and during SRS [12]. However, other studies have not supported this finding with nonsignificant differences in outcomes either with drug discontinuation or not before and during SRS [13]. We also prefer continuing SRL during CK-HFRT as the effect of RT will slowly increase in years and it is not logical to leave the patient with acromegaly untreated for so long until RT becomes fully effective.

In our cohort, 10 (32.3%) patients developed pituitary insufficiency due to CK-HFRT at a median of 62 months follow-up (Table 5). In CK-HFRT studies 2% to 33% pituitary insufficiency rates have been reported at relatively short (three to five years) duration of follow-up [14,15]. Gamma Knife stereotactic radiosurgery (high radiation dose with a single 20 Gy to 35 Gy fraction) in patients with acromegaly cohorts reported similar rates (26% to 34%) of pituitary insufficiency with CK-HFRT series [16]. Pituitary insufficiency rates in both GK-SRS and CK-HFRT studies, high precision conformal treatments and dose fractionation have been related to the protection of pituitary gland functions [17].

We detected optic neuropathy in nine (29%) patients before CK-HFRT of which three (9.7%) improved following irradiation. None of our patients developed RION following hypofractionated radiotherapy (Table 5). In single-fraction high-dose GK-SRS studies for pituitary adenomas, the rate of RION has been reported to be up to 5% [18]. We suggest using fractionated radiotherapy with high-precision techniques to further protect the optic pathway from the side effects of high-dose irradiation.

The risk of radiation-induced secondary brain tumor (RISBT) following RT for pituitary adenoma has been reported to be 2% in 20 years by Brada et al. in 1993. They also reported 0.2% brain radionecrosis at 10 years [19]. A recent large cohort study in Britain confirmed their findings, reporting a 1.9% excessive risk of RISBT in 20 years in patients with pituitary adenoma or craniopharyngioma treated with RT (4%) compared to patients with the same diagnosis who have not been treated with RT (2.1%). This result has shown an increased risk of RISBT development is less than expected. The median latent period following RT has been reported to be 8.3 years for the development of malignant and 17.7 years for benign RISBT [20]. The risk of developing RISBT can be estimated to be even less with GK-SRS and CK-HFRT compared with conventional RT as the irradiated brain volume with these precise RT techniques would be much less than the irradiated volume with conventional RT. None of our patients have developed RISBT or brain radionecrosis during a median of 62-month follow-up.

All three deaths in our study group of 31 patients with acromegaly were related to cardiovascular diseases at 47, 68, and 92 months following CK-HFRT. The crude mortality rate (9.68%) in our study group was 1.7 times higher than the crude mortality rate (5.58%) in Turkey [21]. Mortality data from international registries of acromegaly reported 850 deaths over a total of 8500 patients (10%). The predominant causes of death are cardiovascular diseases and malignancies. Two main trends in acromegaly mortality have been reported first a decrease in cardiovascular deaths while cancer deaths have increased, and second, a general decline in mortality over time. They hypothesized that these trends are related to improved surgery, decreased RT, and new drug treatments [22]. However, in our series, all three deaths have been related to cardiovascular morbidity of treatment-resistant acromegaly and in patients who have been treated with suppressive drugs for four to eight years after CK-HFRT. We generally expect RISBT and cerebrovascular events to limit the use of RT in the treatment of particularly benign diseases such as acromegaly. The increase in malignancy-related deaths in patients with acromegaly cannot be related to RT because observed malignancy-related deaths in patients with acromegaly have been reported as colorectal, thyroid, breast, gastric, and urinary cancers, which can be related to uncontrolled acromegaly rather than RT [23].

There have been two studies before this study reporting their results solely on patients with acromegaly treated with CK-HFRT (Table 7). In these studies and our study, 105 patients with acromegaly have been treated with CK-HFRT. Iwata et al. [15] reported 60% ER rate, and 1.9% pituitary insufficiency caused by CK-HFRT in 52 patients with acromegaly at median 60 months follow-up. Sala et al. found 40.9% CD, 59.1% ER rates, and 36.3% pituitary insufficiency caused by CK-HFRT in 22 patients with acromegaly at a median 43.2 months follow-up [24]. They also reported a higher Biologically Effective Dose (BED) of CK-HFRT in cured patients compared with BED of CK-HFRT in patients with uncontrolled acromegaly (median 163 Gy3 vs 111 Gy3). The cure was achieved in a median of 50 months, and CK-HFRT was effective and safe in the treatment of patients with acromegaly [24]. In our study BED levels of patients with ER vs patients with uncontrolled disease were not significantly different. We report 22.4% CD, 86.7% ER, and 32.3% pituitary insufficiency caused by CK-HFRT in 31 patients with persistent acromegaly at a median of 62 months follow-up. Our lower CD and higher ER rates compared with other CK-HFRT studies most probably depend on our study group which consists of patients with persistent acromegaly. We also report mortality in three out of 31 (9.7%) patients, all related to cardiovascular disease. A common feature of CK-HFRT studies has been the perfect protection of the optic pathway without any reported permanent RION. This does not mean that RION does not occur with the use of CK-HFRT, but by fractionation of irradiation pituitary adenoma can be controlled below the tolerable biologically effective dose for the optic nerve.

**Table 7:** Patients with acromegaly treated with CyberKnife stereotactic hypofractionated radiotherapy D/fx: Dose per fraction; CD: Cured disease; ER: Endocrine remission; NA: Not applicable.

Study	N	D/fx	Median follow-up (months)	CD (%)	ER (%)	Pituitary insufficiency (%)	Radiation-induced optic neuropathy (%)	Mortality (%)
Iwata 2016	52	21Gy/3fx (n=41) 25Gy/5fx (n=11)	60.0	17.0	60.0	1.9	0	0/52 (0%)
Sala 2018	22	20-30 Gy/1fx (n=14) 18-24 Gy/2fx (n=5) 18-24 Gy/3fx (n=2) 27.5Gy/5fx (n=1)	43.2	40.9	59.1	36.3	0	0/22 (0%)
This study 2023	31	21Gy/3fx (n=15) 25Gy/5fx (n=7) 30Gy/10fx (n=4) 20Gy/5fx (n=5)	62.0	22.4	86.7	32.3	0	3/31 (9.7%)

Consensus on the multidisciplinary approach and type of RT in the treatment of patients with acromegaly has been partially achieved to date [25]. Boström et al. suggest risk-adapted RT in patients with acromegaly [26]. They considered patients with pituitary adenoma being >2 mm away from optic pathway and /or PTV <4 ccm as low risk and treated with median 20 Gy (BED=120) (15 Gy - 36 Gy) SRS while pituitary adenomas ≤ 2 mm in distance from the optic pathway and/or PTV ≥ 4 ccm has been considered as high risk and treated with median 54 Gy (BED=78.3) (40 Gy - 55.8 Gy) SRT or 5 x 7 = 35 Gy (BED=96.25) HFRT. They reported 23% cure, 46% pituitary insufficiency and 3% permanent optic neuropathy at a median of eight years follow-up. In a similar way, we irradiated with 10 x 3 Gy (BED=52.5) HFRT the whole content of sella turcica together with bilateral cavernous sinuses when indicated, 5 x 4 Gy (BED=40) from a limited volume for secondary and 3 x 7 Gy (BED=57.75) or 5 x 5 Gy (BED=56.25) for limited volume primary treatments. Acromegaly has been biochemically controlled at a median of 29 months, 44.9% at two years, and 86.7% at five years. Patients have been cured 13.2% at two years and 22.4% at five years. At median 62 months follow-up 10 (32.3%) patients developed pituitary insufficiency while none of the patients suffered ≥G2 permanent RION.

The probability of achieving ER in a shorter time with a higher dose has been studied with GK-SRS in patients with acromegaly. In 31 patients with secretory pituitary adenomas, of which 11 were patients with acromegaly marginal and maximum doses of 35 Gy and 70 Gy, respectively to median 0.7 cm^3^ treatment volume resulted in a median of 18 months to achieve ER in 71% of the patients with 32% pituitary insufficiency. This median time of 18 months to ER was shorter than the median time of approximately 24 months to ER in patients with secretory pituitary adenomas treated with standard GK-SRS doses of 20 Gy to 24 Gy. However, seven patients relapsed in the median 21 months following GK-SRS and relatively smaller adenomas have been chosen for high-dose GK-SRS [27].

Generally higher doses have been used to control functioning pituitary adenomas compared to nonfunctioning pituitary adenomas. Gamma Knife stereotactic radiosurgery or CK-HFRT with higher BED compared with conventionally fractionated RT has been shown to normalize the IGF-I levels in patients with acromegaly with a higher probability and in a shorter time but not at a significant level [28,29]. We found not inferior CD rates, even better ER rates, and acceptable toxicity with standard dose CK-HFRT in patients with persistent acromegaly. 

Considering the side effects of RT, what should be the treatment approach in patients with persistent acromegaly? First, PT options must be used if suppressive drugs are effective with high doses and in combination. SRL ligand dose increase in partial responders, addition of cabergoline if IGF1 persists slightly above age-adjusted limit, and if the patient is diabetic preference of adding pegvisomant should be considered. Therefore, in patients with uncontrolled acromegaly who have already been treated with prolonged PTs and several incomplete surgeries, effective and safe RT options must be considered. Conformality and fractionation of RT will decrease the side effects. CyberKnife hypofractionated radiotherapy with both of these properties presents one of the best RT options in the treatment of patients with persistent acromegaly. In our patients with acromegaly, the addition of CK-HFRT to surgery and PT resulted in similar CD and ER rates to SRS and similar rates of pituitary insufficiency to CRT studies. A striking feature of our study has been the perfect protection of optic pathway with no permanent optic neuropathy diagnosed for 62 months median follow-up of 31 patients with persistent acromegaly.

## Conclusions

CyberKnife stereotactic hypofractionated radiotherapy, as an adjunct to PT, provides a viable treatment option for patients with persistent acromegaly following unsuccessful surgeries. The therapy results in substantial ER rates and tumor control while minimizing the risk of permanent radiation-induced optic neuropathy. However, the decision to administer CK-HFRT should be individualized, considering the patient's overall condition and treatment history.
